# Editorial: Multi-limbed membrane guanylate cyclase cellular signaling pathways

**DOI:** 10.3389/fnmol.2023.1185637

**Published:** 2023-04-12

**Authors:** Rameshwar K. Sharma, Kailash N. Pandey, Teresa Duda, Clint L. Makino

**Affiliations:** ^1^The Unit of Regulatory and Molecular Biology, Research Divisions of Biochemistry and Molecular Biology, Salus University, Elkins Park, PA, United States; ^2^Department of Physiology, Tulane University School of Medicine, New Orleans, LA, United States; ^3^Department of Pharmacology, Physiology and Biophysics, Boston University Chobanian & Avedisian School of Medicine, Boston, MA, United States

**Keywords:** receptor guanylate cyclase, cyclic GMP, ROS-GC, GC-A, GCAP, RD3, retina, mutant mouse

A single molecule was discovered that changed the existing field of cellular signal transduction (reviewed by Sharma and by Duda and Sharma in Chapters 1, 2). The membrane guanylate cyclase (MGC) *via* its seven-limbed structure controlled the entire physiological processes of cardiovasculature, endocrine and sensory neurons; and paved the way that indicated that it may be locked also with the perception of atmospheric conditions. This Research Topic narrates the saga of this molecule that is indispensable for all living beings, from silk pupae to Homo sapiens.

Unlike its predecessors, cyclic AMP and IP_3_, MGC signaling systems consist of a single protein; yet their multi-limbs with diverse molecular structures control various physiological functions. Sharma and Duda and Sharma (Chapters 1, 2) narrate the history, from 1963 up-to-date, of the efforts involved in building on small projects, brick by brick, and its emergence from the chasm of disbelief, through continuous work. The foundational study was the establishment of the first ACTH-modulated limb. It became the structural-physiological template for the future ones. It demonstrated that a common evolutionary link exists between the generation of plant steroids, cardenolides, and the mammalian steroid hormones (see Figure 1 in Sharma, Chapter 1). In the evolutionary ladder, a change occurs. The mammals develop an extra adrenocorticotropic hormone (ACTH)-dependent branch locked with the primordial cholesterol formation. The branch is a sensor of the ACTH signal, generated in the tiniest gland of the brain, the pituitary. The signal is transmitted selectively to the adrenal fasciculata cells; these, from the stored cholesterol synthesized from mevalonic acid, generate the steroid hormone cortisol/corticosterone. In contrast, plants transform cholesterol into cardenolides. Outstandingly, in both cases, the targeted site of cholesterol is its side chain: cleavage in mammals and rearrangement in plants. Notably, the initial biosynthetic steps from mevalonic acid to cholesterol stay almost identical in both the cardenolides and in the rat adrenal mitochondria, only the last biosynthetic steps differ, transformation of the (the 20S)-20-hydroxycholesterol to pregnenolone and then to progesterone and to cortisol/corticosterone. In common, in both kingdoms, the cleavage of the cholesterol sidechain occurs between C-20 and C-22. Cyclic GMP and Ca^2+^ are the sole physiological co-messengers of ACTH.

More recent progress regarding the novel mechanisms of MGC activation, cellular signaling, molecular modeling of structural determinants, and physiological and pathophysiological functions (Figure 1) is presented in this Research Topic in the form of two additional reviews, two brief research reports, and seven original research articles. Chapters 3–6 and 13 are devoted to the MGC receptors for natriuretic factors, GC-A and GC-B, whereas chapters 7–12, to GC-E and GC-F in retinal photoreceptors.

**Figure 1:**
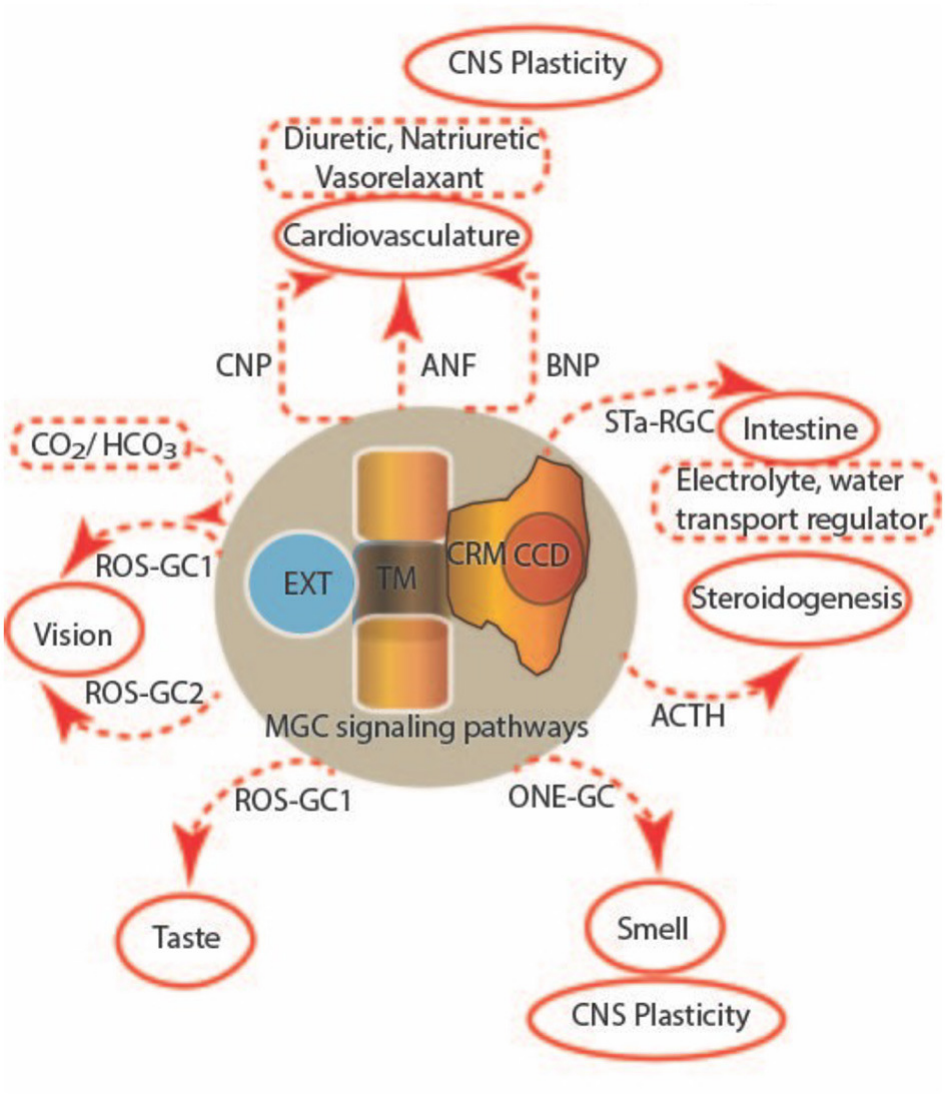
Multi-limbed membrane guanylate cyclase signaling. Seven subfamily forms of membrane guanylate cyclases, encoded by distinct genes, perform a multitude of functions. These uniquely designed surface receptors are all single-pass transmembrane proteins that detect hormones and other extracellular chemicals at an extracellular domain (EXT), translate the signal across a transmembrane domain (TM) to a catalytic regulatory module (CRM) and a catalytic core domain (CCD) to regulate cGMP synthesis. Together, they control steroidogenesis, cardiovasculature, sensory neurons in vision, taste and smell, the intestine, skeletal growth, and CNS plasticity.

Pandey (Chapter 3) reviews the generation and use of genetically engineered animals, including gene-targeted (gene-knockout and gene-duplication) and transgenic mutant mouse models to study the varied roles and pleiotropic functions of GC-A in intact animals. This chapter also provides a chronological development of the biochemical, molecular, physiological, and pathophysiological functions of this MGC, including signaling pathways, genomics, and gene regulation in both normal and diseased states. Egbert et al. (Chapter 4) describe the development of genetically modified mouse lines in which GC-A or GC-B were tagged with a hemagglutinin epitope for tissue localization of the MGCs or had putative phosphorylation sites substituted with glutamates, to mimic the negative charge of the phosphorylated forms so that their regulatory function could be explored. In Chapter 5, Otto and Potter show that vicinal glutamate substitutions mimic phosphorylation more closely than single glutamates in GC-A, and then identify the true regulatory phosphorylation sites. Calis et al. (Chapter 6) provide intriguing findings that GC-A may be linked to long term potentiation in the hippocampus and auditory neural gain through the balanced actions of MR/GR steroid receptors.

GC-E and GC-F in the retinal rods and cones are orphan receptors; no ligands are known to bind their extracellular domains. However, they interact intracellularly with neuronal calcium sensor proteins (e.g., GCAPs), RD3 and bicarbonate. Ames (Chapter 7) reviews control of GC-E activity and proposes a two-state concerted model in which the dimeric MGC allosterically switches between active (R-state) and inactive (T-state) conformational states with distinct quaternary structures. Binding of Ca^2+^-free/Mg^2+^-bound GCAP1 activates GC-E by shifting the balance to the R-state, whereas Ca^2+^-bound GCAP1 and/or RD3 inhibit the cyclase by maintaining the T-state. Geva et al. (Chapter 8) describe how an axial gradient of bicarbonate within the rod outer segment creates spatial inhomogeneity in the rates of cGMP synthesis that affects variability in photon response amplitude and kinetics. Surprisingly, bicarbonate imposes markedly different effects in the rods of salamander and toad, increasing variability in the former while decreasing it in the latter. Bicarbonate appears to dampen the primary phototransduction cascade at one end of the toad rod outer segment by an unknown mechanism. This novel effect of bicarbonate solves a mystery as to how toad vision is able to function effectively in extremely dim light. Adhikari et al. (Chapter 9) describe how bicarbonate more profoundly affects flash response kinetics and maximal response amplitude in mouse rods, at a lower concentration, when compared to amphibian rods. In addition, bicarbonate expands the dynamic range over which rods respond to dimmer flashes in mouse, whereas the range is extended to brighter flashes in amphibians. Caruso et al. (Chapter 10) refined a previously developed, space-resolved biophysical model of rod phototransduction by incorporating several mechanisms that play significant roles in shaping the rod response under high illumination levels: the function of RGS9 in shutting off G protein, and Ca^2+^ dependences of: rhodopsin phosphorylation, cGMP binding to the light-regulated ion channel, and two MGC activities. The updated model explains how depletion of RGS9 complex with very bright flashes affects response saturation behavior and further predicts what would happen with flashes so bright that they activate all the available PDE. During a screening for murine models of human ocular disorders, Naggert et al. (Chapter 11) discovered a mutation in GC-E that leads to an early loss of cone photoreceptor function. Rods survive initially because they also express GC-F, but they eventually succumb to a progressive degeneration, which may be relevant to understanding disease pathology in LCA. During evolution, whole genome duplications resulted in as many as three MGCs in vertebrate visual photoreceptors, regulated by as many as eight GCAP Ca^2+^ sensors. Observing that most mammals retain two MGCs and up to three GCAPs, whereas reptiles and birds pair a single MGC with up to five GCAPs, Gesemann and Neuhauss (Chapter 12) hypothesized that evolutionary forces may drive diversity in GCAPs to offset the reduced number of MGCs.

Based on amino acid sequence homology, Chen et al. (Chapter 13), suspected that RD3 (retinal degeneration type 3), thought to suppress ROS-GC activity until it was localized properly within the rod photoreceptor, might serve an analogous function on natriuretic peptide MGCs in other cells. Indeed, Chen et al. show RD3 expression in tissues expressing GC-A and GC-B: retina, cerebellum, hippocampus, neocortex and olfactory bulb, and inhibition of GC-A and GC-B activities by RD3 in biochemical assays.

Despite the ample progress that has been made, there is still much to discover regarding the molecular structures of MGCs, how they activate upon ligand binding or are controlled by regulatory proteins, and their expanding roles in cellular signaling, physiological and pathophysiological functions. Future investigations should also lead to exciting and innovative strategies toward the prevention, diagnosis, and treatment of a wide range of diseases.

## Author contributions

RKS, TD, KNP, and CLM contributed to the writing and editing of the manuscript. Figure provided by RKS. All authors contributed to the article and approved the submitted version.

